# Correction: Identifying inpatient hospitalizations with continuous electroencephalogram monitoring from administrative data

**DOI:** 10.1186/s12913-024-11896-y

**Published:** 2024-11-12

**Authors:** Marta Fernandes, M. Brandon Westover, Sahar F. Zafar

**Affiliations:** 1 Department of Neurology, Massachusetts General Hospital (MGH), 55 Fruit Street, Boston, MA 02114 USA; 2 Harvard Medical School, Boston, MA USA; 3 Department of Neurology, Beth Israel Deaconess Medical Center, Boston, MA USA

**Correction to: BMC Health Services Research 23, 1234 (2023)**

**https://doi.org/10.1186/s12913-023-10262-8**

In this article the ‘Competing interests’ statement was incorrectly given as “The authors declare no competing interests.” but should have been “Dr. M. Brandon Westover has private equity as co-founder of Beacon Biosignals and receives compensation for consulting and scientific advisory roles. The other authors declare no competing interests.”

The original article has been updated.

