# Supplementary material for: Identifying inpatient hospitalizations with continuous electroencephalogram monitoring from administrative data
Source: BMC Health Serv Res. 2023 Nov 10;23:1234. doi: 10.1186/s12913-023-10262-8 (PMC10636942; doi:10.1186/s12913-023-10262-8)
Supplement: Supplementary file 1 — Supplementary Material 1 [file 12913_2023_10262_MOESM1_ESM.docx]

## Appendix

Contents

[Appendix 1](#_Toc122947744)

[A.1. Study data 2](#_Toc122947745)

[**Table A1.** International Classification of Diseases (ICD) and Current Procedural Terminology (CPT) codes used to define diagnoses and procedures covariates. 2](#_Toc122947746)

[**Table A2.** Modeling input covariates distribution for the hospital inpatient admissions in the study cohort. 3](#_Toc122947747)

[A.2. Hyperparameter tunning 6](#_Toc122947748)

[**Table A3.** Hyperparameters selected for the extreme gradient boosting model trained with all features in 10-fold cross validation. 6](#_Toc122947749)

[A.3. Modeling results 7](#_Toc122947750)

[**Figure A1**. Curves for the extreme gradient boosting model trained with all covariates evaluated on the test set: (a) area under the receiver operating characteristic curve, and (b) area under the precision recall curve. cEEG – acute inpatient admissions class; EMU – epilepsy monitoring unit admissions class. 7](#_Toc122947751)

[**Figure A2**. Curves for the extreme gradient boosting model trained with all covariates, except for the admission type covariates and discharge disposition to home or self-care, evaluated on the test set: (a) area under the receiver operating characteristic curve, and (b) area under the precision recall curve. cEEG – acute inpatient admissions class; EMU – epilepsy monitoring unit admissions class. 8](#_Toc122947752)

[**Table A4.** Confusion matrices of the extreme gradient boosting model trained with all covariates, evaluated in the testing set for different thresholds. 9](#_Toc122947753)

[**Table A5.** Modeling performance [95% confidence intervals] of the extreme gradient boosting model trained with all covariates, except for the admission type covariates and discharge disposition to home or self-care, evaluated in the testing set. 10](#_Toc122947754)

[**Figure A3.** Shapley Additive exPlanations (SHAP) values of the top 20 features in the extreme gradient boosting model trained with all covariates. Positive SHAP values further from zero represent higher impact for the acute inpatient admissions class (cEEG) while negative SHAP represent higher impact for the epilepsy monitoring unit admissions (EMU) class. 11](#_Toc122947755)

### A.1. Study data

#### **Table A1.** International Classification of Diseases (ICD) and Current Procedural Terminology (CPT) codes used to define diagnoses and procedures covariates.

| **Variable** | **ICD-10 codes** | **CPT code** |
| --- | --- | --- |
| **Diagnosis** |  |  |
| Epilepsy, seizures or convulsions | G40.*, R56.9 | - |
| Stroke | I60-I69 | - |
| TBI | S00-S09 | - |
| **Daily laboratory values** | 005009 | 80048, 85007, 85025, 85027 |
| **Procedures** |  |  |
| Abdomen/Pelvis CT scan | BW241ZZ, BW24YZZ, BW24ZZZ, BW20ZZZ, BW200ZZ, BW201ZZ, BW20YZZ, BW2GZZZ, BW2G0ZZ, BW2G1ZZ, BW2GYZZ, BW21ZZZ, BW210ZZ, BW211ZZ BW21YZZ, BW25ZZZ, BW250ZZ, BW251ZZ, BW25YZZ | 74176-74178 |
| Arterial line | 03HC3DZ | 36620 |
| Chest X-ray | BW03ZZZ | 71045-71048 |
| Head CT scan | BW28ZZZ, BW280ZZ, BW281ZZ, BW28YZZ, BW29ZZZ, BW290ZZ, BW291ZZ, BW29YZZ | 70450, 70460, 70470, 70480-70482, 70496 |
| LP | G97.1 | 62270 |
| LTM EEG | 4A10X4Z | 95707, 95710, 95713, 95716-95726, 95951, 95956 |
| MRI | B030ZZZ | 70541, 70544-70548, 70551-70553, 70557, 70559, 73221, 73223, 73721, 73723 |
| Mechanical ventilation | 5A1935Z, 5A1945Z, 0BH17EZ | 31500, 94002-94004, 94375, 94640, 94642, 94660, 94727 |
| TTE | Z51.81 | 93308 |
| Tube feed order | - | B4034-B9999, 43752 |

CT – computerized tomography; EEG – electroencephalogram; LP – lumbar puncture; LTM – long-term electroencephalographic monitoring; MRI – Magnetic resonance imaging; TBI – Traumatic brain injury; TTE – Transthoracic echocardiogram.

#### **Table A2.** Modeling input covariates distribution for the hospital inpatient admissions in the study cohort.

| **Covariate** | **EMU**  **(N =912)** | **cEEG**  **(N = 9,871)** | **Hospital admissions**  **(N = 10,783)** |
| --- | --- | --- | --- |
| **Age ^(a)^, (years, mean (SD))** | 42 (17.9) | 60.0 (17.6) | 59 (18.3) |
| **Female sex, N (%)** | 504 (55.3) | 4,518 (45.8) | 5,022 (46.6) |
| **Hispanic ethnicity, N (%)** | 68 (7.5) | 741 (7.5) | 809 (7.5) |
| **Race, N (%)** |  |  |  |
| White | 719 (78.8) | 7,418 (75.1) | 8,137 (75.5) |
| Black or African American | 52 (5.7) | 901 (9.1) | 953 (8.8) |
| Asian | 29 (3.2) | 343 (3.5) | 372 (3.4) |
| **Type of admission, N (%)** |  |  |  |
| Emergency | 159 (17.4) | 6,743 (68.3) | 6,902 (64.0) |
| Urgent | 93 (10.2) | 2,053 (20.8) | 2,146 (20.0) |
| Elective | 660 (72.4) | 1,069 (10.8) | 1,729 (16.0) |
| **Discharge disposition, N (%)** |  |  |  |
| Deceased | 8 (0.9) | 1,889 (19.1) | 1,897 (17.6) |
| Home or Self Care | 754 (82.7) | 2,058 (20.8) | 2,812 (26.1) |
| **Diagnosis, N (%)** |  |  |  |
| TBI | 5 (0.5) | 693 (7.0) | 698 (6.5) |
| Stroke | 14 (1.5) | 1,493 (15.1) | 1,507 (14.0) |
| Epilepsy, seizures or convulsions | 451 (49.5) | 6,425 (65.1) | 6,876 (63.8) |
| **Primary diagnosis, N (%)** |  |  |  |
| TBI | 2 (0.2) | 257 (2.6) | 259 (2.4) |
| Stroke | 4 (0.4) | 366 (3.7) | 370 (3.4) |
| Epilepsy, seizures or convulsions | 111 (12.2) | 967 (9.8) | 1,078 (10.0) |
| **Daily laboratory values acquired, N (%)** | 537 (58.9) | 9,590 (97.2) | 10,127 (93.9) |
| **Procedures, N (%)** |  |  |  |
| Abdomen/Pelvis CT scan | 42 (4.6) | 2,869 (29.1) | 2,911 (27.0) |
| Arterial line | 58 (6.4) | 1,626 (16.5) | 1,684 (15.6) |
| Chest X-ray | 139 (15.2) | 6,913 (70.0) | 7,052 (65.4) |
| Head CT scan | 174 (19.1) | 6,372 (64.6) | 6,546 (60.7) |
| LP | 22 (2.4) | 839 (8.5) | 861 (8.0) |
| MRI | 209 (22.9) | 5,660 (57.3) | 5,869 (54.4) |
| Mechanical ventilation | 38 (4.2) | 3,781 (38.3) | 3,819 (35.4) |
| TTE | 0 (0.0) | 1,099 (11.1) | 1,099 (10.2) |
| Tube feed order | 75 (8.2) | 2,459 (24.9) | 2,534 (23.5) |
| **Medications, N (%)** |  |  |  |
| Cefepime | 35 (3.8) | 4,125 (41.8) | 4,160 (38.6) |
| Ceftriaxone | 158 (17.3) | 3,669 (37.2) | 3,827 (35.5) |
| Dexmedetomidine | 43 (4.7) | 3,423 (34.7) | 3,466 (32.1) |
| Dobutamine | 0 (0.0) | 270 (2.7) | 270 (2.5) |
| Dopamine | 1 (0.1) | 213 (2.2) | 214 (2.0) |
| Enoxaparin | 780 (85.5) | 6,357 (64.4) | 7,137 (66.2) |
| Epinephrine | 123 (13.5) | 5,083 (51.5) | 5,206 (48.3) |
| Heparin | 56 (6.1) | 4,938 (50.0) | 4,994 (46.3) |
| Midazolam | 120 (13.2) | 3,721 (37.7) | 3,841 (35.6) |
| Nicardipine | 67 (7.3) | 2,655 (26.9) | 2,722 (25.2) |
| Norepinephrine | 22 (2.4) | 1,705 (17.3) | 1,727 (16.0) |
| Phenylephrine | 141 (15.5) | 4,499 (45.6) | 4,640 (43.0) |
| Piperacillin | 0 (0.0) | 154 (1.6) | 154 (1.4) |
| Piperacillin/tazobactam | 19 (2.1) | 1,131 (11.5) | 1,150 (10.7) |
| Propofol | 176 (19.3) | 5,891 (59.7) | 6,067 (56.3) |
| Vancomycin | 176 (19.3) | 5,601 (56.7) | 5,777 (53.6) |
| Vasopressin | 4 (0.4) | 1,897 (19.2) | 1,901 (17.6) |
| **Medications ^(b)^, (number, median [IQR])** | 1 [1, 2] | 6 [2, 9] | 5 [1, 8] |
| **Procedures ^(b)^, (number, median [IQR])** | 0 [0, 1] | 3 [2, 5] | 3 [1, 5] |
| **LOS (days, median [IQR])** | 6 [3, 9] | 10 [5, 21] | 9 [5, 19] |

cEEG – acute inpatient admissions class; CT – computerized tomography; EMU – epilepsy monitoring unit class; IQR – interquartile range; LOS – length of stay; LP – lumbar puncture; LTM – long term electroencephalographic monitoring class; MRI – magnetic resonance imaging; N – number of hospital admissions; SD – standard deviation; TBI – traumatic brain injury; TTE – transthoracic echocardiogram. **^(a)^** Age at encounter. **^(b)^** The medications or procedures are the ones listed on this table.

### A.2. Hyperparameter tunning

The following extreme gradient boosting parameters were tuned {set of values}: number of trees {100, 150, 200, 250, 300, 350}; maximum tree depth {2, 3, 4, 5}; learning rate {0.01, 0.05, 0.06, 0.07, 0.08, 0.09, 0.1}; gamma, which specifies the minimum loss reduction required to make a split {0, 1, 5}; percentage of features selected to build a tree {30, 40, 50, 60, 70, 80}; percentage of hospital admissions in train selected to build a tree {80, 90, 100}; and the “warm_start” parameter was varied between True/False, to reuse the solution of the previous call to fit as initialization (True) or erase the previous solution (False). All data preprocessing and modeling were performed in Python version 3.7.

#### **Table A3.** Hyperparameters selected for the extreme gradient boosting model trained with all features in 10-fold cross validation.

| **Hyperparameter** | **Value** |
| --- | --- |
| Number of trees | 150 |
| Tree depth | 3 |
| Sample of features used to build a tree (%) | 60 |
| Sample of hospital admissions used to build a tree (%) | 80 |
| Gamma | 1 |
| Learning rate | 0.07 |
| Warm start | False |

### A.3. Modeling results

#### **Figure A1**. Curves for the extreme gradient boosting model trained with all covariates evaluated on the test set: (a) area under the receiver operating characteristic curve, and (b) area under the precision recall curve. cEEG – acute inpatient admissions class; EMU – epilepsy monitoring unit admissions class.


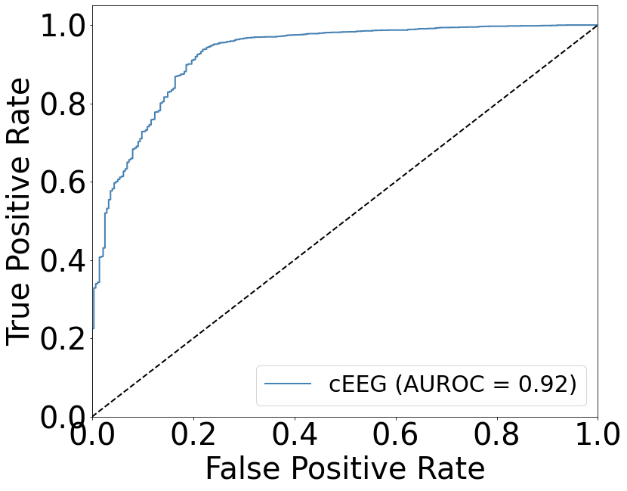

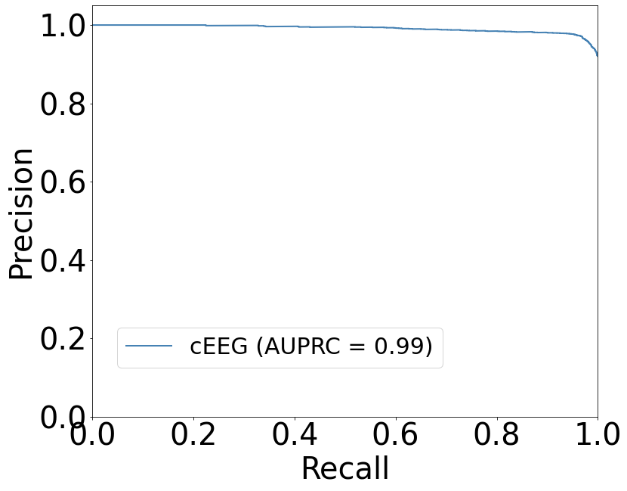


(a) (b)

#### **Figure A2**. Curves for the extreme gradient boosting model trained with all covariates, except for the admission type covariates and discharge disposition to home or self-care, evaluated on the test set: (a) area under the receiver operating characteristic curve, and (b) area under the precision recall curve. cEEG – acute inpatient admissions class; EMU – epilepsy monitoring unit admissions class.


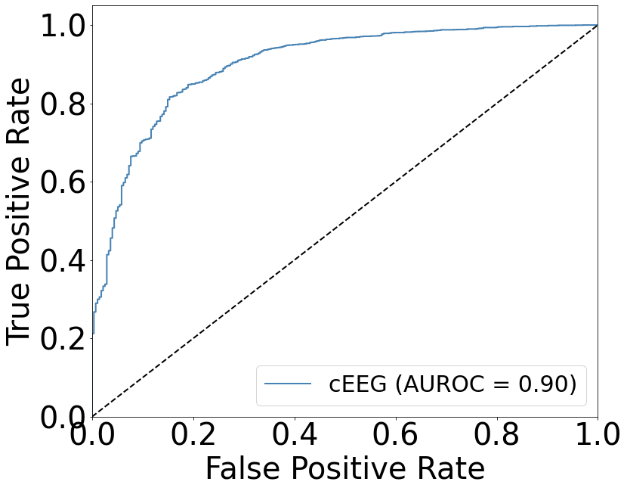

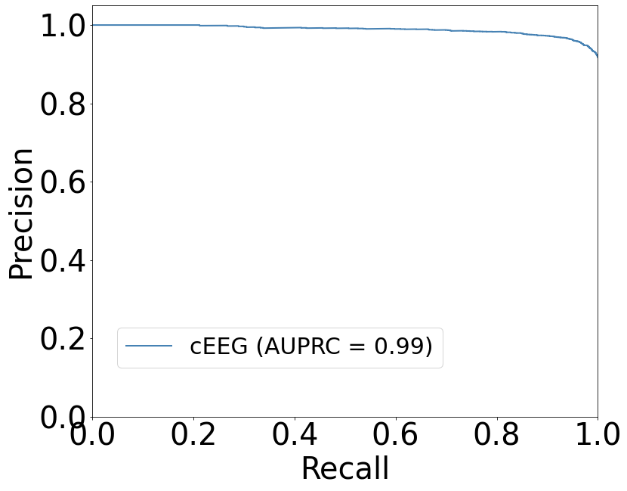


(a) (b)

#### **Table A4.** Confusion matrices of the extreme gradient boosting model trained with all covariates, evaluated in the testing set for different thresholds.

| **PPV (%)** | **Normalization** | **Train set** | **Test set** |
| --- | --- | --- | --- |
| 95 | Sensitivity | 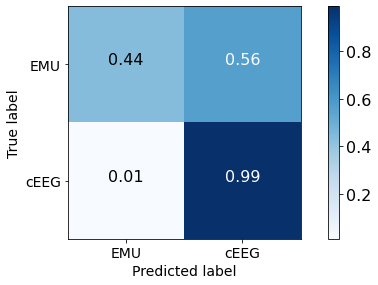 | 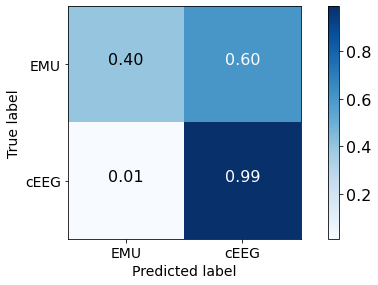 |
|  | PPV | 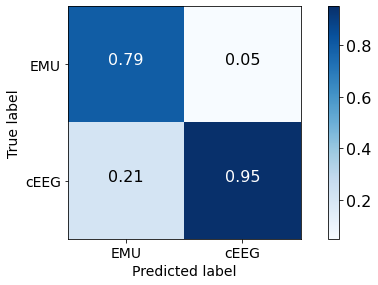 | 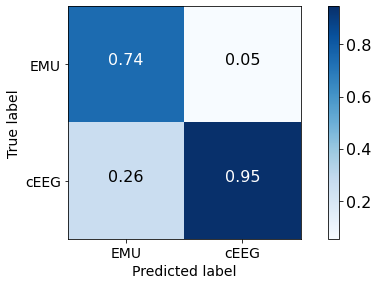 |
| 96 | Sensitivity | 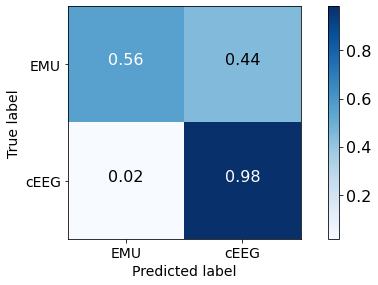 | 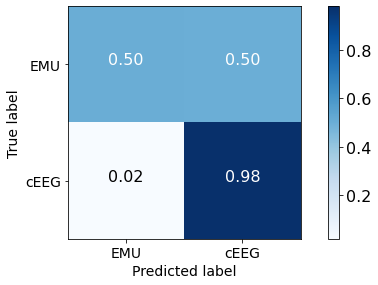 |
|  | PPV | 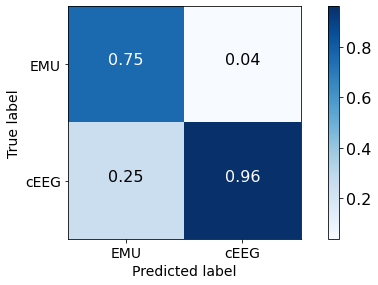 | 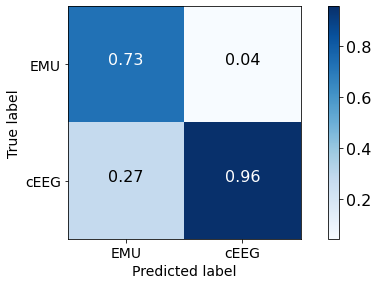 |
| 97 | Sensitivity | 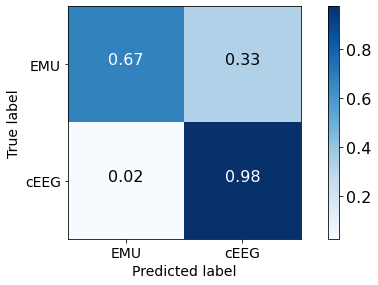 | 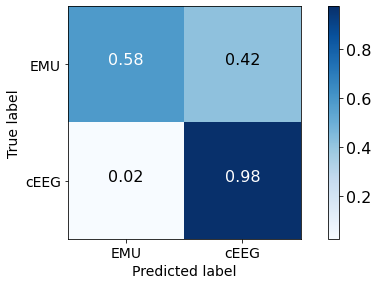 |
|  | PPV | 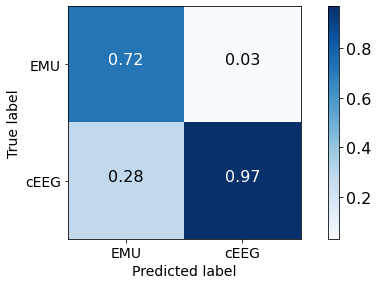 | 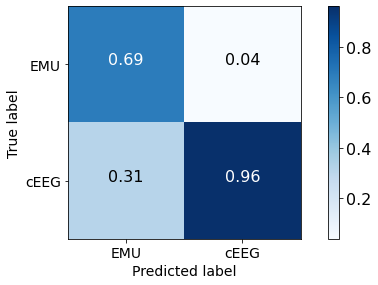 |
| 98 | Sensitivity | 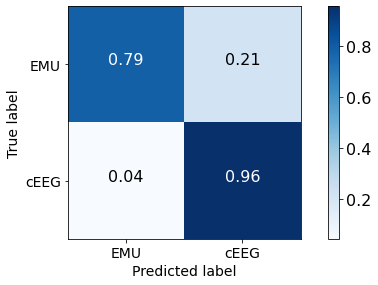 | 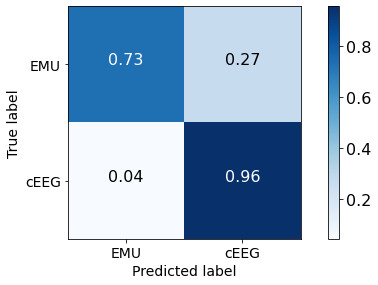 |
|  | PPV | 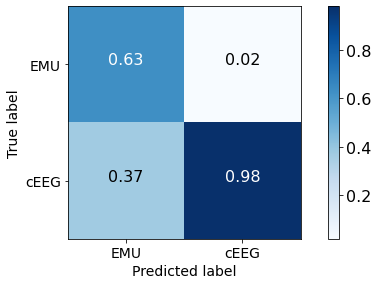 | 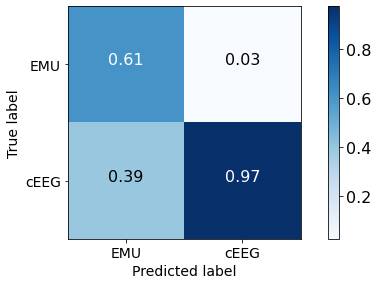 |

cEEG – acute inpatient hospitalizations admissions class; EMU – epilepsy monitoring unit admissions class; PPV – positive predictive value.

#### **Table A5.** Modeling performance [95% confidence intervals] of the extreme gradient boosting model trained with all covariates, except for the admission type covariates and discharge disposition to home or self-care, evaluated in the testing set.

| **Classes** | **AUROC** | **AUPRC** | **Sensitivity** | **PPV** | **NPV** | **Specificity** |
| --- | --- | --- | --- | --- | --- | --- |
| Macro average | 0.90  [0.88-0.92] | 0.98  [0.97-0.98] | 0.70  [0.67-0.73] | 0.79  [0.75-0.83] | 0.70  [0.75-0.83] | 0.70  [0.67-0.73] |
| EMU | 0.90  [0.88-0.92] | 0.56  [0.49-0.62] | 0.58  [0.52-0.64] | 0.53  [0.47-0.58] | 0.96  [0.95-0.97] | 0.95  [0.94-0.96] |
| cEEG | 0.90  [0.88-0.92] | 0.99  [0.98-0.99] | 0.95  [0.94-0.96] | 0.96  [0.95-0.97] | 0.53  [0.47-0.58] | 0.58  [0.52-0.64] |

AUROC – area under the receiver operating characteristic curve; AUPRC – area under the precision-recall curve; cEEG – acute inpatient hospitalizations admissions class; EMU – epilepsy monitoring unit admissions class; NPV - negative predictive value; PPV – positive predictive value.

#### **Figure A3.** Shapley Additive exPlanations (SHAP) values of the top 20 features in the extreme gradient boosting model trained with all covariates. Positive SHAP values further from zero represent higher impact for the acute inpatient admissions class (cEEG) while negative SHAP represent higher impact for the epilepsy monitoring unit admissions (EMU) class.


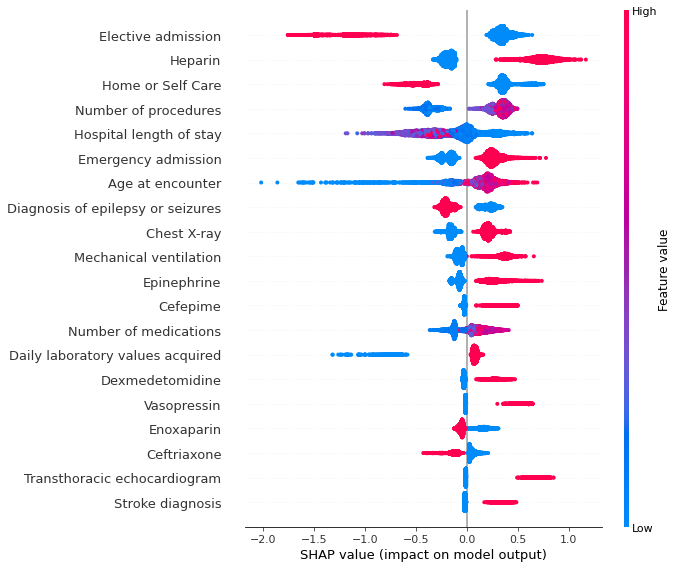


Positive SHAP values further from zero represent higher probability of the cEEG class, on the right side of Figure 3, while negative SHAP, on the left side, represent higher probability of the EMU class. The blue color is associated with lower covariate values, while pink is associated with higher covariate values. Binary covariates are either absent (blue) or present (pink). For numerical covariates, namely age, hospital LOS, and number of distinct procedures and medications, there are intermediate colors since these covariates vary in the range [0, 1]. Each colored dot represents an admission, and each admission has a SHAP value for each covariate. Vertical overlapping dots occur where admissions share the same SHAP values.
